# A Rice Autophagy Gene *OsATG8b* Is Involved in Nitrogen Remobilization and Control of Grain Quality

**DOI:** 10.3389/fpls.2020.00588

**Published:** 2020-06-04

**Authors:** Tian Fan, Wu Yang, Xuan Zeng, Xinlan Xu, Yanling Xu, Xiaorong Fan, Ming Luo, Changen Tian, Kuaifei Xia, Mingyong Zhang

**Affiliations:** ^1^School of Life Sciences, Guangzhou University, Guangzhou, China; ^2^Innovation Academy for Seed Design, Guangdong Provincial Key Laboratory of Applied Botany, Key Laboratory of South China Agricultural Plant Molecular Analysis and Genetic Improvement, South China Botanical Garden, Chinese Academy of Sciences, Guangzhou, China; ^3^State Key Laboratory of Crop Genetics and Germplasm Enhancement, Nanjing Agricultural University, Nanjing, China; ^4^Center of Economic Botany, Core Botanical Gardens, Chinese Academy of Sciences, Guangzhou, China

**Keywords:** autophagy, *OsATG8b*, nitrogen recycling, rice, seed quality

## Abstract

Enhancing nitrogen (N) use efficiency is a potential way to reduce excessive nitrogen application and increase yield. Autophagy is a conserved degradation system in the evolution of eukaryotic cells and plays an important role in plant development and stress response. Autophagic cores have two conjugation pathways that attach the product of autophagy-related gene 8 (ATG8) to phosphatidylethanolamine (PE) and ATG5 to ATG12, respectively, which then help with vesicle elongation and enclosure. Rice has six *ATG8* genes, which have not been functionally confirmed so far. We identified the rice gene *OsATG8b* and characterized its role in N remobilization to affect grain quality by generating transgenic plants with its over-expression and knockdown. Our study confirmed the autophagy activity of OsATG8b through the complementation of the yeast autophagy-defective mutant *scatg8* and by observation of autophagosome formation in rice. The autophagy activity is higher in *OsATG8b*-OE lines and lower in *OsATG8b*-RNAi than that in wild type (ZH11). ^15^N pulse-chase analysis revealed that *OsATG8b*-OE plants conferred higher N recycling efficiency to grains, while *OsATG8b*-RNAi transgenic plants exhibited lower N recycling efficiency and poorer grain quality. The autophagic role of *OsATG8b* was experimentally confirmed, and it was concluded that OsATG8b-mediated autophagy is involved in N recycling to grains and contributes to the grain quality, indicating that OsATG8b may be a potential gene for molecular breeding and cultivation of rice.

## Introduction

Nitrogen (N) is one of the most limiting nutrients for crop yield. Increasing N utilization efficiency (NUE) is not only important for increasing yield and reducing production cost but also for avoiding environmental pollution and keeping agriculture sustainable (Good et al., 2004; Masclaux-Daubresse et al., 2010). Therefore, it is very important to find effective genes to improve NUE and yield. Plant N utilization involves complex mechanisms of absorption, translocation, assimilation, and remobilization. Of those steps, N remobilization plays an important role during seed filling (Masclaux-Daubresse et al., 2008, 2010). At the vegetative stages, most N uptake is directed to leaves, in which most proteins are synthesized. During the reproductive stage, leaf proteins degrade rapidly to amino acids and small peptides, which are transported to seeds (Masclaux-Daubresse et al., 2008). N remobilization of cereals in senescent leaves has been shown to account for 50–90% of the grain N content (Kichey et al., 2007). The 26S proteasome/ubiquitin system and autophagy are the two main pathways of protein degradation (Wada et al., 2009; Roberts et al., 2012). Autophagy can degrade proteins, bulk organelles and cytosolic macromolecules with low selectivity and high throughput (Suzuki and Ohsumi, 2007).

Autophagy is a conserved degradation system in the evolution of eukaryotic cells. In the process of autophagy, the cytoplasm and organelles are separated by bilayer vesicles called autophagosomes and transported to vacuoles of yeast and plant cells or lysosomes of animal cells for degradation and recycling (Nakatogawa et al., 2009; Li and Vierstra, 2012; Yoshimoto, 2012). More than 30 autophagy-related genes (ATGs) have been identified in yeast, and 17 of them are necessary for autophagosome formation (Xie and Klionsky, 2007; Yoshimoto, 2012). Recently, orthologs of most yeast core *ATG* genes have been found in *Arabidopsis* and rice (Doelling et al., 2002; Hanaoka et al., 2002; Yoshimoto et al., 2004; Bassham et al., 2006; Xia et al., 2011). ATG8 is one of the core proteins for forming autophagosome. It covalently binds to membrane lipid phosphatidylethanolamine (PE) through a ubiquitin-related binding system (Xie and Klionsky, 2007). ATG8 is a scaffold for membrane expansion and elongation during autophagosome formation (Nakatogawa et al., 2007; Xie et al., 2008). Yeast *ATG8* also participates in the cytoplasm-to-vacuole targeting (CVT) pathway. Vacuole hydrolases, such as the precursor of aminopeptidase 1 (APE1), are selectively transported into the vacuole to produce mature APE1 (Yamaguchi et al., 2010). Unlike yeast, which has a single copy of the *ATG8* gene, plants usually have an *ATG8* family, comprising nine genes in *Arabidopsis* (Yoshimoto et al., 2004), five in maize (Chung et al., 2009), and six in rice (Xia et al., 2011). The different expression patterns of *Arabidopsis ATG8*s suggest that some *ATG8*s possess functional diversity besides possible redundancy (Slavikova et al., 2005).

Like yeast and animals, plant autophagy plays an important role in nutrient recycling under N- and C-starvation conditions (Li and Vierstra, 2012; Ohsumi, 2014). Currently, research on autophagy often focuses on the remobilization of N (Guiboileau et al., 2012, 2013; Xia et al., 2012; Li et al., 2015). Most *Arabidopsis ATG* genes are up-regulated by N-starvation and during leaf senescence (Doelling et al., 2002; Rose et al., 2006). Loss of function of *Arabidopsis* autophagy (*atg5*, *atg7*, *atg10*, and *atg13a atg13b*) caused hypersensitivity to N-limiting conditions in *Arabidopsis* and accelerated senescence even under N-rich conditions (Hanaoka et al., 2002; Phillips et al., 2008; Suttangkakul et al., 2011). Overexpression of *AtATG8f* and *GmATG8c* made *Arabidopsis* more tolerant to both N- and C-starvation (Slavikova et al., 2008; Xia et al., 2012). Autophagy mutants of *Arabidopsis* and maize (*atg5* and *atg7* in *Arabidopsis* and *atg12* in maize) showed reduced seed yield, seed N content, and N remobilization efficiency (NRE) (Guiboileau et al., 2012, 2013; Li et al., 2015). About 50% of the remobilized N of *Arabidopsis* is proven to come from autophagy (Guiboileau et al., 2012). These studies showed that autophagy plays a central role in N remobilization.

Since evidence for the contribution of autophagy to plant physiology largely comes from the study of *Arabidopsis*, little is known about crop autophagy except for maize. Rice is an important cereal crop for the world population, especially in Asia. Currently, little is known about the contribution of autophagy to rice seed quality. Rice *OsATG7* plays a role in NUE at the vegetative stage (Wada et al., 2015), and overexpression of rice gene *osatg8b* confers tolerance to nitrogen starvation and increases yield and nitrogen use efficiency in *Arabidopsis* (Zhen et al., 2019). However, the male sterility of *osatg7* limits research on autophagy-mediated N recycling to grains in rice.

In our study, we functionally analyzed *OsATG8b* in rice. Complementation of a yeast *atg* mutant and subcellular localization analysis demonstrated the role of *OsATG8b* in the autophagy process. In addition, we characterized the role of *OsATG8b* in N remobilization and seed quality by generating transgenic plants with over-expression and knockdown of *OsATG8b*. The phenotypic and ^15^N-partitioning analysis showed that *OsATG8b* plays a role in N remobilization and grain quality. This result may provide strategic guidance for N application in molecular breeding and production of rice.

## Materials and Methods

### Plant Materials and Growth Conditions

From spring to autumn, the *japonica* rice cultivar Zhonghua11 (ZH11) and transgenic plants were grown in a controlled paddy with normal planting. In winter, they were grown in a greenhouse at 28°C with 14-h light and 10-h dark per day. For hydroponic experiments, we used the modified rice nutrient solution of the International Rice Research Institute (IRRI, 1.43 mM NH_4_NO_3_, 0.32 mM NaH_2_PO_4_, 0.51 mM K_2_SO_4_, 1 mM CaCl_2_, 1.65 mM MgSO_4_, 8.9 mM MnSO_4_, 0.5 mM Na_2_MoO_4_, 18.4 mM H_3_BO_3_, 0.14 mM ZnSO_4_, 0.16 mM CuSO_4_, 40 mM FeSO_4_) in a growth room with a 30°C, 14 h light/10 h dark photoperiod (Yoshida et al., 1976). The solution was refreshed every 3-day. For nitrogen treatments, after the plants were germinated in water, they were grown on the IRRI solution for 7 days, and then plants were grown in the IRRI solution supplemented with high nitrogen (HN, 5 mM NH_4_NO_3_) and low nitrogen (LN, 0.2 mM NH_4_NO_3_) for different times.

### Quantitative Real-Time RT-PCR (qRT-PCR)

Total RNA isolation, cDNA synthesis, and qRT-PCR of the rice were performed as previously described (Xia et al., 2011). Relative gene expression was normalized to the expression level of *e-EF-1a* with triplicate repeat. All primers are listed in Supplementary Table S1. qRT-PCR was repeated with three biological replicates, and each sample was assayed in triplicate by PCR.

### Complementation of Yeast *scatg8* Mutants

*OsATG8b* ORF was cloned downstream of promoter *GAL1* of the yeast vector pYES260. Wild-type yeast KVY55 and the *scatg8* mutant KVY5 (MATa *leu2 ura3 trp1 lys2 his3 suc2-△9△atg8::HIS3*) were gifts from Dr. Yoshinori Ohsumi (Tokyo Institute of Technology, Japan). The vector was transformed into *scatg8* according to the LiAc/SS-DNA/PEG TRAFO protocol (Clontech). Yeast were cultured and shaken at 30°C in SC medium supplemented with 0.67% (w/v) YNB (yeast N base without NH_4_SO_4_ and amino acids), 2% (w/v) galactose, 0.5% (w/v) NH_4_SO_4_, and Ura DO Supplement. When the yeast grew to the logarithmic metaphase of growth (OD_600_ = 1), yeast cells were collected by centrifugation, washed, and incubated for another 5 h in 0.67% YNB medium without amino acids, galactose and NH_4_SO_4_ for nutrient deprivation to induce autophagy. The collected cells were used for immunoblotting with anti-APE1 antibody (Santa Cruz); the immunoblot analysis process used was as previously described (Hamasaki et al., 2005).

### Scanning Electron Microscopy

Rice seeds were used for scanning electron microscopy (SEM) analysis. Samples were fixed overnight with 3% glutaraldehyde-sodium phosphate buffer (0.1M) at room temperature and rinsed three times with 0.1M sodium phosphate buffer. The samples were dehydrated through an ethanol series and infiltrated with an isoamyl acetate series. Seeds were then sputter-coated with gold/palladium in six different 30 s pulses (Hitachi JEE-420) and analyzed by scanning electron microscope (Hitachi S-3000N).

### Subcellular Localization of *OsATG8b* Protein Fused With Green Fluorescent Protein Derivatives

*GFP-OsATG8b* and *sGFP-OsATG8b* were constructed to analyze the subcellular localization of OsATG8b in yeast and rice, respectively. For yeast subcellular localization, the fused construct was inserted downstream of promoter *GAL1* in pYES260 vector. For rice subcellular localization, the fused construct was inserted downstream of *35S* promoter (Okano et al., 2008). For root imaging, 7-day seedlings were treated with 1 μM concanamycin A for 6 h at 28°C in darkness, and 5 mm of root from the root tip was cut off for observation. The green fluorescent protein (GFP) fusion protein was analyzed by confocal laser scanning microscope (ZEISS-710 Meta) with a 488-nm exciting wavelength. The thickness of the optical sections (pinhole) was 2.1 μm. The images presented are average projections of 8–20 optical sections.

### Generation of *OsATG8b*-Overexpression and -RNAi Transgenic Plants

To overexpress *OsATG8b*, the full-length CDS of *OsATG8b* was amplified by PCR and was inserted into the intermediate vector pUC18-sGFP. The whole cassette was finally PCR-amplified and inserted into the binary vector pCAMBIA1301 to replace the *GUS* via *Nco* I and *BstE* II. For the construction of the RNAi vector, a 230-bp fragment of the non-conserved 5′ end of *OsATG8b* was amplified with primers OsATG8b-Ri-F and OsATG8b-Ri-R and inserted in vector pTCK303 by *Bam*H I and *Kpn* I for the sense strand and by *Spe* I and *Sac* I for the antisense strand (Wang et al., 2004). These vectors were transformed into *A*. *tumefaciens* EHA105 and then transformed into ZH11 with the Agrobacterium-mediated transformation method (Hiei et al., 1997).

### Antibodies

Antibodies of OsATG8b were made with 6 × His-OsATG8b proteins as the antigen; these were purified using a Ni column (Novagen) and injected directly into rabbits by Beijing ComWin Biotech Co., Ltd.

### Protein Extraction and Immunoblot Analysis

Two-week old seedlings were used for total cell extracts and were ground in liquid N. The powders were extracted with the lysis buffer (25 mM Tris-HCl pH7.5, 1 mM EDTA, 1% Triton X-100, 150 mM NaCl, and Complete Protease Inhibitor Cocktail from Roche). The solution was then centrifuged at 13,000*g* for 20 min at 4°C, and the supernatant was used as total protein. The supernatant was run by SDS-PAGE with or without 6M urea and then transferred to nitrocellulose filter membranes for immunoblot analysis. The membranes were blocked and then incubated with mouse GFP antibodies (Santa Cruz) at a dilution of 1:1,000, while rabbit serum of OsATG8b was diluted by 1:500. All results came from three independent plant materials.

### ^15^N-Labeling and Determination of ^15^N Content

Rice plants were grown in IRRI solution in a greenhouse with 16-h light/8-h dark cycling. At 40 days after germination (DAG), plants were labeled with ^15^N for 5-day by adding 10 atom% excess Na^15^NO_3_ to the IRRI solution. The plants were then washed thoroughly with distilled H_2_O and transferred in the field for further growth. For ^15^N uptake measurements, thirteen plants of each genotype were harvested 2-day after ^15^N labeling. The ^15^N-labeled plants were further grown in the field to maturity, and grains and remains were separated for N recycling assessment. A dry weight (DW) of each sample was assayed for ^15^N and total N content using an isotope ratio mass spectrometer coupled with an N elemental analyzer (IsoPrime100, Elemental Scientific, United States). The ^15^N content of each sample was calculated as a % of total N, which was calculated as atom% or A%_sample_ = 100 × (^15^N)/(^15^N + ^14^N) (Li et al., 2015).

### NUE and N Recycling Efficiency (NRE) Calculations

Factors of calculation for NUE and NRE were estimated through the procedure provided by Guiboileau et al. (2012) and Li et al. (2015). The HI (harvest index) for yield evaluation was defined as the DW_grain_/(DW_remain_ + DW_grain_). The N harvest index (NHI) for assessing grain filling with N was calculated as N%_grain_ × DW_grain_/(N%_remain_ × DW_remain_ + N%_grain_ × DW_grain_). NUE was then calculated as the NHI/HI ratio, and NUE values of different genotypes were compared. The efficiency of N recycling to grains was shown by ^15^NHI (^15^N harvest index), which was calculated by (A%_grain__s_ × N%_grain__s_ × DW_grain__s_)/[(A%_remain_ × N%_remain_ × DW_remain_) + (A%_grains_ × N%_grains_ × DW_grains_)]. The ^15^NHI:HI ratio was used to compare the NRE of different transgenic plants. ^15^N-labeling data were compiled from three biological replicates involving five plants for each genotype.

### Quantification of Soluble Proteins and Starch

Total protein concentration and starch content were determined as described previously (Masclaux-Daubresse et al., 2002; Carlsson et al., 2011). Quantification data were compiled from three biological replicates involving 40 seeds from five plants for each genotype.

## Results

### *OsATG8b* Restores Autophagy Activity in Yeast *scatg8* Mutant

Six *OsATG8*s have been identified in the rice genome (Xia et al., 2011). The ATG8 phylogenetic tree generated from amino acid sequences showed that plant ATG8s are clustered into two main subgroups. Subgroup I covers most of the plant *ATG8* family members, comprising *OsATG8a*, *b*, and *c*. Subgroup II covers 1–3 plant *ATG8* family members from each species, containing *OsATG8d*, *e*, and *f* (Supplementary Figure S1). The existence of two subgroups may imply specific functions to each, besides possible redundancy. To explore the relationship between N remobilization derived by autophagy and rice grain quality, we analyzed the expression of *OsATG8s* in developing endosperm by searching the Rice Expression Profile Database (RiceXpro)^1^ and found that only *OsATG8b* expression increased with endosperm development compared with *OsATG8a* and *OsATG8c* (Supplementary Figure S8). These data indicated that the *OsATG8b* may be a potential rice ATG8 gene in grain filling, and it was chosen for further analyses. OsATG8b is encoded by a single gene (Os04g0642400) in rice. It is a soluble protein of 119 amino acids, with a predicted molecular mass of 13.7 kD and pI of 8.78. OsATG8b shares 81.8% amino acid identity with yeast ScATG8, 71.4% identity with human HsGABARAP, and 86.9% identity with *Arabidopsis* AtATG8a (Supplementary Figure S2A). Like other ATG8 proteins, OsATG8b has a conserved Gly residue at the C-terminus for PE conjugation (Supplementary Figure S2A). The result of 3D model prediction revealed that OsATG8b protein contains an N-terminal helical domain, two hydrophobic pockets named the W-site and the L-site, and a C-terminal ubiquitin-like domain, similar to yeast ScATG8 (Supplementary Figures S2A,B) (Noda et al., 2010). This implies that OsATG8b may have the autophagic function, similar to yeast ScATG8.

**FIGURE 1:**
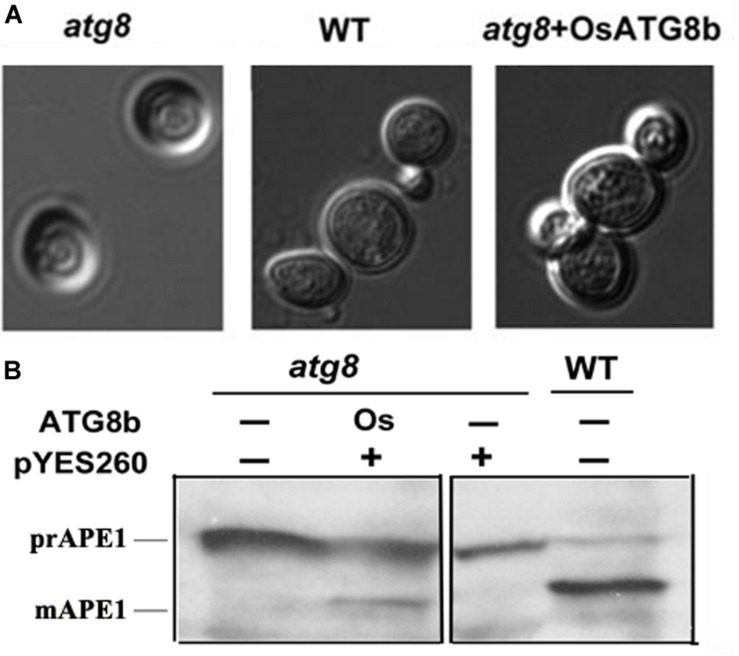
Functional complementation of yeast *atg8* mutant by *OsATG8b*. *OsATG8b* cDNA was cloned into the plasmid pYES260 and expressed in yeast *atg8* mutant KVY5 strain. **(A)** *OsATG8b* restores normal cell morphology of the yeast *scatg8* mutant under N starvation. Abnormal morphology of *scatg8* yeast cell (left), compared with normal cell of wild-type (WT) yeast (middle), and *scatg8* yeast transformed with *OsATG8b* (right). **(B)** API protein identification by Western blot. Yeast cells were grown to mid-log phase to be collected and washed, incubated in the presence of 2% galactose to induce *OsATG8b* expression, and then incubated for another 5 h in nutrient deprivation medium and harvested for protein extraction. Proteins were then resolved by SDS-PAGE followed by immunoblotting with anti-APE1 antibody. prAPE1, precursor of aminopeptidase 1; mAPE1, mature aminopeptidase 1.

To verify the autophagic function of *OsATG8b*, we investigated whether *OsATG8b* rescues defects of *ATG8*-deficient (*scatg8*) yeast KVY5 (Kirisako et al., 1999). *OsATG8b* cDNA containing the entire ORF was driven by the yeast *GAL1* promoter in a plasmid (pYES260) and expressed in *scatg8* yeast. *OsATG8b* can rescue the abnormal cell morphology of the *scatg8* yeast under N starvation (Figure 1A). In yeast, the precursor amino-peptidase1 (prAPE1) was delivered to the vacuole for processing into mature APE1 (mAPE1) through the Cvt/autophagy pathway (Yamaguchi et al., 2010). Thus, we monitored the protein levels of both prAPE1 and mAPE1 after 5 h of starvation. Both wild-type yeast and *scatg8* cells complemented with *OsATG8b* accumulated mAPE1. In contrast, mAPE1 was detected in neither *scatg8* cells nor the *scatg8* cells transformed with the empty vector (Figure 1B). This suggests that prAPE1 was delivered to the vacuole and processed to mAPE1 in *scatg8b* yeast when *OsATG8b* was expressed in these cells. These results confirmed the autophagic activity of OsATG8b and showed that OsATG8b is a functional homolog of yeast ScATG8.

### *OsATG8b* Expression Is Induced by N- and C-Starvation

**FIGURE 2:**
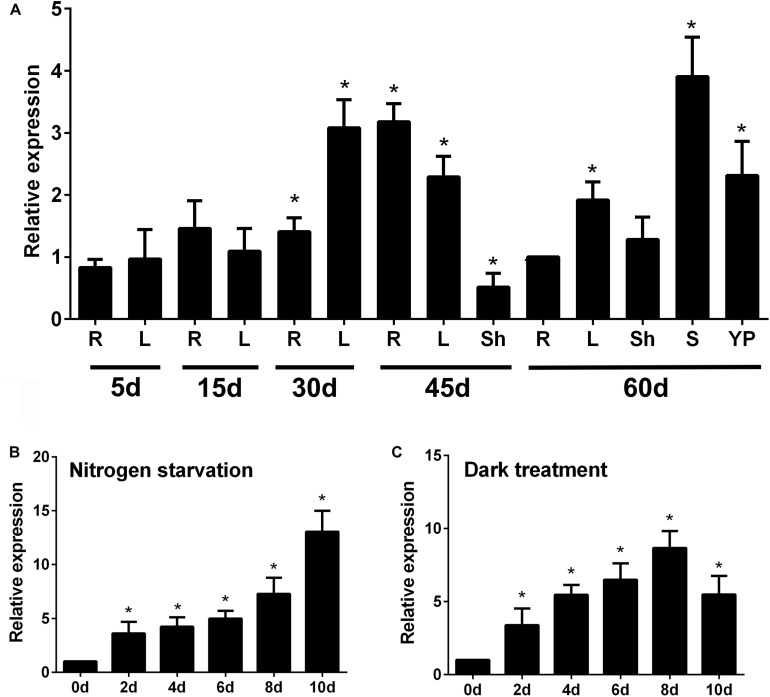
Expression patterns of *OsATG8b* in developing tissues of rice by qRT-PCR analysis. **(A)** Expression of *OsATG8b* in developing tissues. Total RNA was isolated from roots €, sheaths (Sh), young panicles (YP), stems (S), and leaves (L) at different growth stages. *OseEF-1a* was used as an internal reference. **(B,C)** Expression profiles of *OsATG8b* in an N-starvation solution **(B)** and in darkness **(C)**. Total RNA was isolated from 5-leaf seedlings treated for 2, 4, 6, 8, and 10-day with N-starvation and darkness. Error bars indicate standard deviations of independent biological replicates (*n* = 3). One asterisk (*p < 0.05, *t*-test) represents significant difference.

To determine the spatial and temporal expression pattern of *OsATG8b*, we employed qRT-PCR to examine *OsATG8b* expression. qRT-PCR analysis showed that *OsATG8b* transcripts accumulated in all studied organs, including roots, stems, leaves, leaf sheaths, and panicles at different growth stages (Figure 2A). The results showed that *OsATG8b* transcript levels were higher in roots of plants at 45 days after germination (DAG) than in those of plants at other growth stages. At 60 DAG, *OsATG8b* transcript was relatively abundant in stems, leaf, and panicle (Figure 2A). The expression level of *OsATG8b* was also examined under N deficiency and darkness treatment for C starvation, respectively (Figures 2B,C). *OsATG8b* transcript level increased in response to both N deficiency and darkness treatment. When rice seedlings were subjected to the N-free treatment, the expression level of *OsATG8b* gradually increased, peaking at 10-day after treatment application. Similarly, darkness treatment rapidly induced a roughly three-fold increase in *OsATG8b* expression within 2-day after treatment. Taken together, these results suggest that *OsATG8b* may play a crucial role in regulating multiple developmental processes and in response to nutrient stresses.

### GFP-OsATG8b Is Localized to Autophagosomes

**FIGURE 3:**
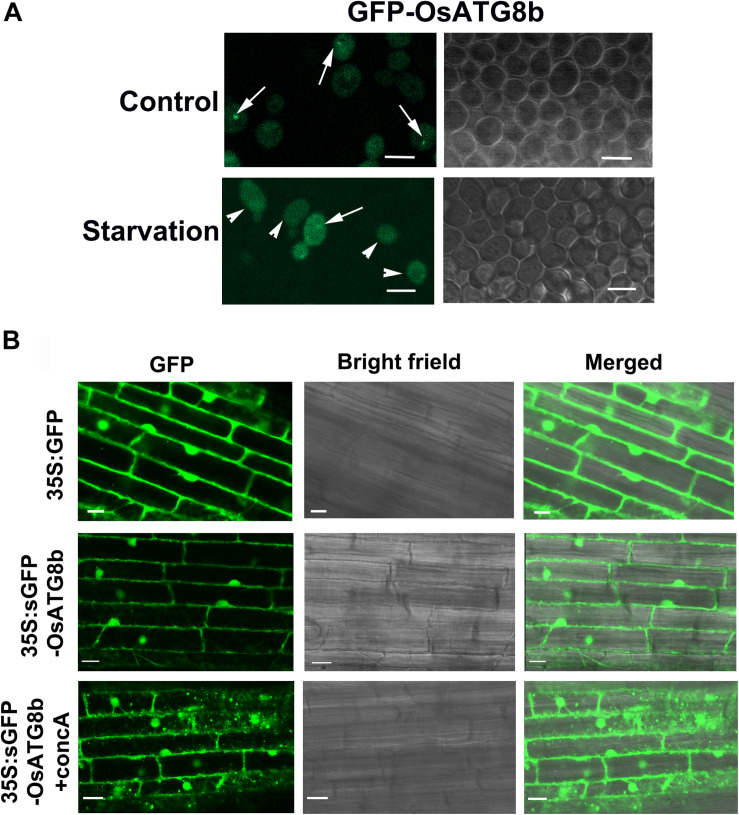
Subcellular localization of OsATG8b. **(A)** Subcellular localization of GFP-OsATG8b in yeast cells. Yeast cells were grown to mid-exponential phase and then incubated in the presence of 2% galactose to induce *GFP-OsATG8b* expression. Cells grown in both the control and starvation media were visualized by confocal laser scanning microscope. Arrows indicate the pre-autophagosomal structure (PAS), and arrowheads indicate GFP within vacuoles. Bars = 5 μm. **(B)** Subcellular localization of sGFP-OsATG8b in roots of transgenic rice. Fresh root samples of transgenic rice plants expressing *35S:sGFP-OsATG8b* were excised and observed immediately (0 h) and after 6-h treatment with 1 μM concanamycin A. The region at approximately 5 mm from the root tip was observed in a laser scanning confocal microscope. Simultaneously obtained sGFP fluorescence images and DIC images are shown. Bars = 10 μm.

To determine whether OsATG8b is conjugated onto the autophagosome membrane and completed or delivered into the vacuole, GFP was fused to its N-terminus and transformed into *scatg8* yeast cells. Under control conditions, GFP-OsATG8b was mainly localized in the cytosol with punctate distribution, whereas after starvation, it accumulated within the vacuole of yeast (Figure 3A). These data suggest that OsATG8b may be localized to the autophagosomes of cytosol under the control conditions and translocate from the cytosol to the vacuole in an autophagy-dependent manner after starvation in yeast. To further verify the above result in rice, *sGFP-OsATG8b* was also transiently expressed in rice protoplasts, but the data showed that the sGFP-OsATG8b fusion protein was localized to the membrane, cytoplasm, and nucleus (Supplementary Figure S3), similar to the free sGFP control. To further confirm sub-cellular localization, transgenic rice expressing s*GFP-OsATG8b* were generated under the control of *35S* promoter (Figure 3B). The 5 mm of the roots from the tip were cut off and immediately observed by LSCM. In *sGFP-OsATG8b*, GFP fluorescence was detected in the cytoplasm and nucleus; however, after 6 h of incubation in darkness with concanamycin A (an inhibitor of vacuolar H^+^-ATPase) to help in the observation of autophagic bodies through increasing vacuolar pH (Ishida et al., 2008; Izumi et al., 2015), many vesicles with a strong GFP signal and the spread of a faint GFP signal were observed (Figure 3B). These results indicate that the sGFP-OsATG8b-labeled puncta located in autophagosomes and the sGFP-OsATG8b can be used to visualize the progression of autophagy in rice, and overexpression of *OsATG8b* could increase the autophagic activity. Immunoblot analysis using proteins isolated from either ZH11 or transgenic *sGFP* and *sGFP-OsATG8b* rice plants showed that the OsATG8b antibodies recognized the endogenous as well as the GFP fusion proteins (Supplementary Figures S4A,B). Meanwhile, we performed an sGFP-ATG8 processing assay by the levels of free GFP moiety in anti-GFP immunoblots. After entering into the vacuole, sGFP-ATG8 is digested and releases free GFP, which is in a soluble and relatively stable form during autophagic body turnover (Suttangkakul et al., 2011). This free vacuolar GFP accumulates to a higher level when autophagy accelerates, so it represents the transport of ATG8 to vacuoles. Since OsATG8b antibody can also recognize OsATG8a and c (Supplementary Figure S9), the endogenous OsATG8(a/b/c) band of ZH11 and G1 (Supplementary Figure S4B) is very weak, but in *OsATG8b*-OE, the OsATG8(a/b/c) band is very strong, indicating that OsATG8b is overexpressed. These results indicated that the OsATG8 has already conjugated onto the autophagosome membrane and is able to be completed or delivered into the vacuole (Supplementary Figure S4A), which further confirms the autophagic activity of OsATG8b.

### Knockdown of *OsATG8b* Expression Affects Root Growth at Grain Germination Stage

**FIGURE 4:**
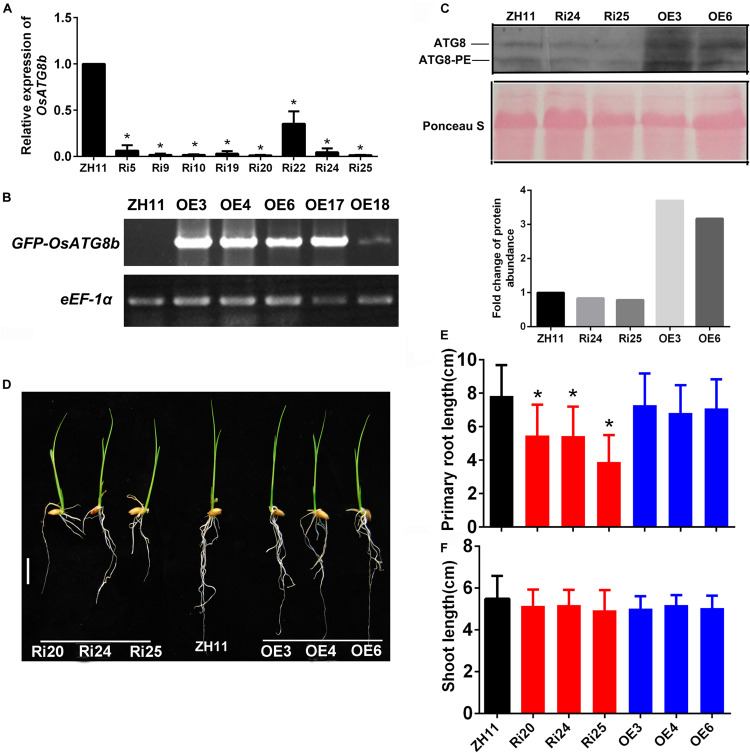
*OsATG8b* affects root growth at seed germination stage. **(A,B)** Expression levels of *OsATG8b* in flag leaves of *OsATG8b*-RNAi (Ri) **(A)** and *OsATG8b*-overexpression (OE) lines **(B)**, measured by qRT-PCR and semi-qRT-PCR analyses, respectively. *OseEF-1a* was used as an internal reference. Error bars indicate standard deviations of independent biological replicates (*n* = 3). One asterisk (*p < 0.05, *t*-test) represents significant difference. **(C)** Immunoblot detection of the OsATG8a/b/c and OsATG8a/b/c lipidation level in 14 days-after-germination shoots of ZH11, *OsATG8b*-RNAi, and *OsATG8b*-OE lines. Total protein extracts were subjected to SDS-PAGE in the presence of 6M urea and immunoblotted with OsATG8b antibody. The fold change of ATG8a/b/c-PE was quantified using Image J software v1.45. **(D–F)** Seedlings **(D)** and statistical analysis of root **(E)** and shoot length **(F)** of *OsATG8b*-RNAi and *OsATG8b*-OE lines and ZH11 at 7-day after germination in water. Bar = 1 cm. Three biological replicates, each containing 80 plants, were used for data analysis. Results are the mean ± SD from 80 plants. *p < 0.05 (*t*-test): significantly different from ZH11. No asterisks mean no significant difference.

To investigate the function of *OsATG8b*, *OsATG8b* over-expression (*OsATG8b*-OE), and RNA-interference (*OsATG8b*-RNAi), transgenic lines were generated. RT-PCR analysis showed that *OsATG8b* expression increased in flag leaves of *OsATG8b*-OE lines and decreased in flag leaves of *OsATG8b*-RNAi lines (Figures 4A,B). The *OsATG8b*-RNAi construct was targeted specifically to the non-conserved 5′ end (Supplementary Figure S5A) of *OsATG8b* outside the ubiquitin domain to avoid interference with other OsATG8 proteins. Three of the *OsATG8b*-RNAi lines (Ri20, Ri24, and Ri25) and three of the *OsATG8*-OE lines (OE3, OE4, and OE6) were selected for subsequent analysis. In order to observe the effect of altered *OsATG8b* expression on *OsATG8a* and *OsATG8c*, we detected the expression of *OsATG8a* and *OsATG8c* in the shoots and roots of the transgenic rice seedling at four-leaf stage (Supplementary Figures S5B,C). The results showed that there is no significant difference in *OsATG8a* or *OsATG8c* transcript level among ZH11, the *OsATG8*-OE lines, and the *OsATG8b*-RNAi lines.

To confirm whether autophagic activities are altered in the *OsATG8b*-RNAi and *OsATG8b*-OE lines, we examined the ATG8a/b/c autophagic activities in 14 days-after-germination shoots of *OsATG8b*-RNAi, *OsATG8b*-OE, and ZH11 lines using OsATG8b antibodies (Figure 4C). The bands of ATG8 and ATG8-PE respectively represent the sum of OsATG8a/b/c or OsATG8a/b/c-PE (Figure 4C), since OsATG8b antibody can also recognize OsATG8a and OsATG8c (Supplementary Figure S9). The immunoblot analysis showed that the levels of OsATG8a/b/c-PE (representing the forming or completed autophagosomes) and cytosolic OsATG8a/b/c form were remarkably increased in *OsATG8b*-OE lines compared with ZH11 lines, and the quantified results showed that there is a slight decrease (about 17–20%) in them in *OsATG8b*-RNAi compared with in ZH11. Because *OsATG8a/c* expression does not change in these transgenic rice (Supplementary Figures S5B,C), these changes to the immunoblot bands should represent the changes of OsATG8b and OsATG8b-PE in *OsATG8b*-OE and *OsATG8b*-RNAi (Figure 4C). These results indicated that the autophagic activity is higher in *OsATG8b*-OE lines and may be a little lower in *OsATG8b*-RNAi than that in ZH11.

When the role of OsATG8b in growth at the vegetative stage was analyzed, we observed that the roots of 7-day-old *OsATG8b*-RNAi seedlings were much shorter than those of ZH11 and *OsATG8b*-OE lines (Figures 4D,E) when germinated in water. To reveal how the N level affects autophagy in rice, growth of *OsATG8b*-RNAi and *OsATG8b*-OE lines was measured under low nitrogen (LN, 0.2 mM NH_4_NO_3_) and high nitrogen (HN, 5 mM NH_4_NO_3_) for 30- or 60-day. Under LN and HN levels, the *OsATG8b*-RNAi and *OsATG8b*-OE lines exhibited a relatively normal phenotype and a similar growth rate when compared with ZH11 at 30 (Supplementary Figures S6A–D) or 60 DAG (Supplementary Figures S6E–H). Neither root nor shoot length showed any significant difference among these lines (Supplementary Figures S6C,D,G,H). These data may indicate that knocking down *OsATG8b* affects root growth at the stage of seed germination.

### *OsATG8b* Affects Grain Yield and Grain Quality in Rice

**FIGURE 5:**
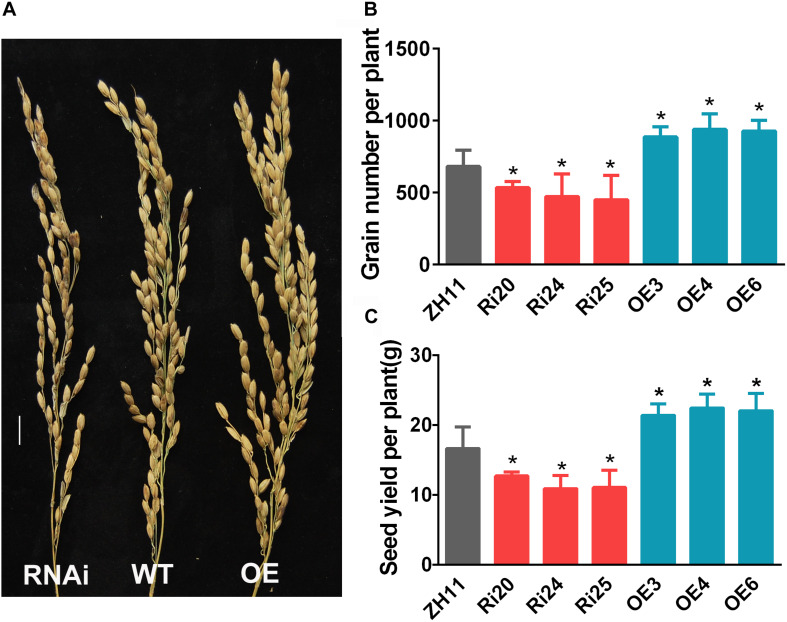
*OsATG8b* affects rice grain yield. *OsATG8b*-RNAi (Ri) and *OsATG8b*-OE (OE) lines and ZH11 were grown in the paddy field with normal fertilizer. **(A–C)** Panicle architecture **(A)**, grain number **(B)**, and grain yield **(C)** per plant of *OsATG8b-*RNAi and *OsATG8b*-OE lines and ZH11. Bar = 1 cm. Three biological replicates, each containing 80 plants, were used for data analysis. Results are the mean ± SD from 80 plants. *p < 0.05 (*t*-test): significantly different from ZH11.

The phenotypes of *OsATG8b*-RNAi and *OsATG8b*-OE rice at the reproductive stage were investigated in the paddy field under normal N conditions. Previous studies have shown that the autophagy-defective rice mutant *osatg7* displayed complete sporophytic male sterility. However, *OsATG8b*-RNAi and *OsATG8b*-OE plants produced healthy pollen grains and could be fertilized normally. The statistical results showed that grain number and grain yield per plant increased in *OsATG8b*-OE plants but decreased in *OsATG8b*-RNAi ones compared with ZH11 plants (Figure 5). These data indicate that *OsATG8b* may be involved in grain development and yield.

**FIGURE 6:**
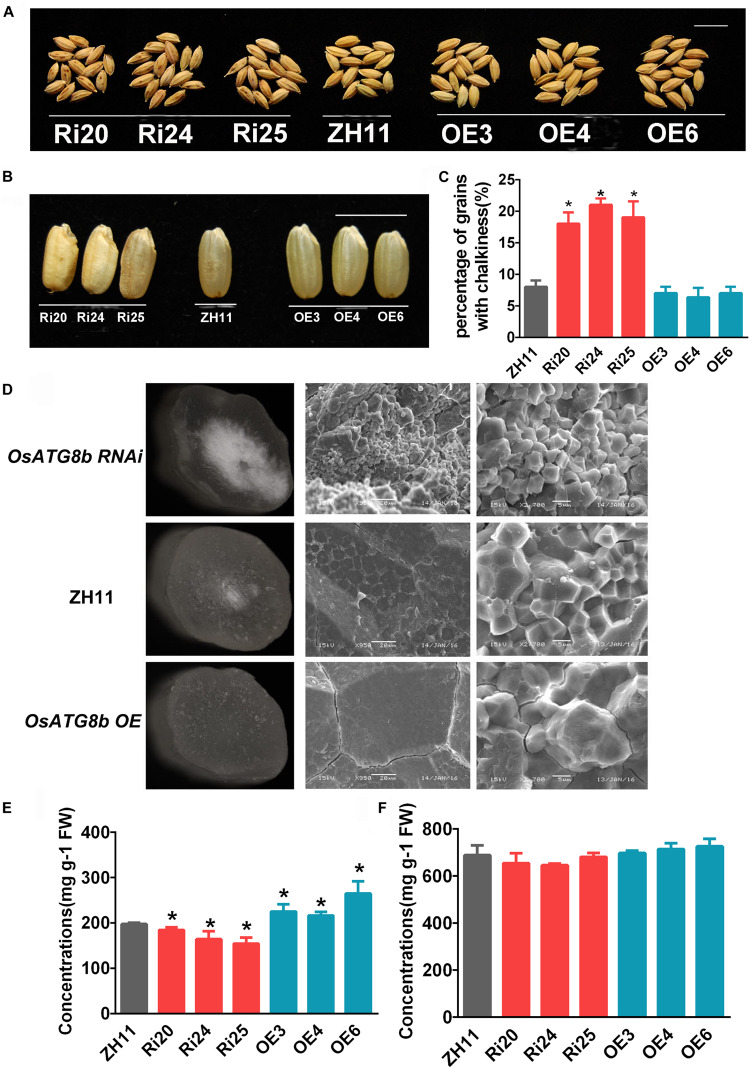
*OsATG8b*-mediated autophagy affects grain quality in rice. *OsATG8b*-RNAi (Ri) and *OsATG8b*-OE (OE) lines and ZH11 were grown in a paddy field under normal growth conditions. **(A)** Seed grains. *OsATG8b*-RNAi lines produced grains with brown spotted hulls. Bar = 1 cm. **(B)** Hulled *OsATG8b*-RNAi rice lines showed a chalky endosperm phenotype. Bar = 1 cm. **(C)** Percentage of hulled rice with chalkiness. **(D)** Scanning electron micrographs of cracked mature caryopses of rice grains under different magnifications. Endosperms of *OsATG8b*-RNAi lines had small, loosely packed starch granules, which differed markedly from the large, tightly packed starch granules of ZH11 and *OsATG8b-*OE lines. **(E)** Soluble protein concentration in grains. **(F)** Starch concentration in grains. Three biological replicates, each containing 40 seeds from five plants, were used for data analysis. Results are the mean ± SD from five plants. *p < 0.05 (*t*-test): significantly different from ZH11 **(C,E,F)**.

The grains of *OsATG8b*-RNAi have a brown-spotted hull and contain chalky endosperm (Figures 6A,B). This showed that it produced poor quality seeds. The percentage of hulled rice with chalkiness was higher in *OsATG8b*-RNAi lines than in ZH11 (Figure 6C). SEM revealed that there are many loosely packed and small starch granules in the endosperm of *OsATG8b*-RNAi, which differed from the large and tightly packed starch granules in ZH11 (Figure 6D). Conversely, endosperm starch granules of *OsATG8b*-OE and ZH11 grains seemed larger and tighter (Figure 6D). Compared with ZH11, soluble protein content in *OsATG8b-*RNAi lines was lower, while that in *OsATG8b-*OE lines was higher (Figure 6E). However, starch content showed no significant difference among those lines (Figure 6F).

### *OsATG8b* Affects N Recycling to Grains

**FIGURE 7:**
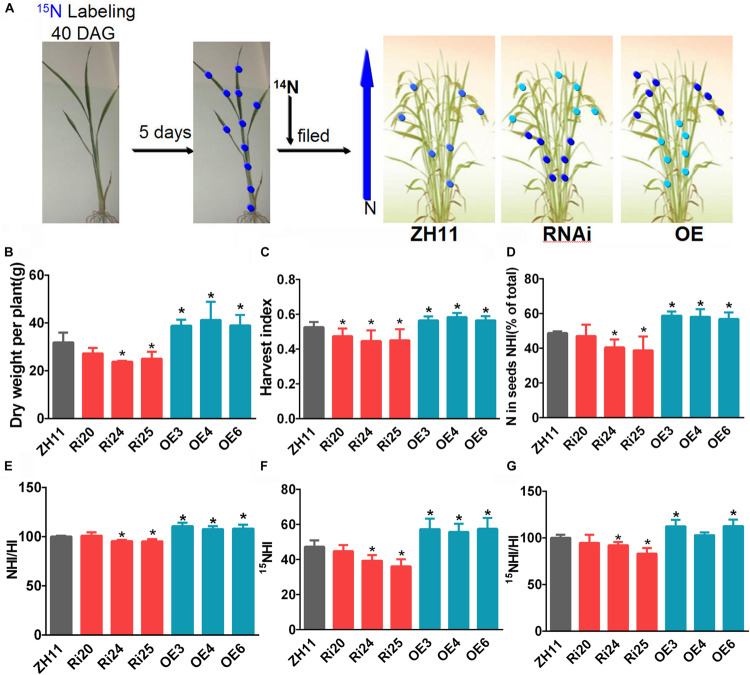
*OsATG8b*-mediated autophagy significantly affects N recycling efficiency (NRE). *OsATG8b*-RNAi (Ri) and *OsATG8b*-OE (OE) lines and ZH11 were grown in IRRI solution with ^14^NO_3_^–^ supplementation until 40-day after germination and then labeled by a 5-day pulse of ^15^NO_3_^–^ and subsequently grown in normal conditions to mature stage. After grain filling, ^15^N and the ^14^N/^15^N ratio were analyzed by isotopic ratio mass spectrometry. **(A)** Overview of ^15^N labeling and subsequent N partitioning. The deeper the blue of the dot, the higher the N content. **(B)** Biomass accumulation as measured by dry weight (DW) per plant. **(C)** Harvest index (HI) as measured by ratio of DW of grains to DW of the aboveground plant parts. **(D)** Nitrogen harvest index (NHI) as measured by the partitioning of total plant N in grains. **(E)** NHI:HI ratio as an estimate of N use efficiency. **(F)** Partitioning of total ^15^N in grains. **(G)** ^15^NHI:HI ratio as an indicator of N recycling efficiency. Three biological replicates, each containing five plants, were used for data analysis. Results are the mean ± SD from five plants. *p < 0.05 (*t*-test): significantly different from ZH11.

To investigate whether *OsATG8b* plays a role in N recycling to grains in rice, we performed a pulse-chase assay with ^15^NO_3_^–^, as previously conducted with *Arabidopsis* (Masclaux-Daubresse and Chardon, 2011; Guiboileau et al., 2012). ^15^N and the ^14^N/^15^N ratio were measured (Figure 7A). Plant dry weight (DW) was higher in *OsATG8b*-OE lines and lower in *OsATG8b*-RNAi lines than in ZH11 (Figure 7B). This is similar to what was observed in *Arabidopsis* mutants (*atg5*, *atg7*) (Doelling et al., 2002; Guiboileau et al., 2012). HI, an important productivity indicator (Yang and Zhang, 2010), was lower in *OsATG8b*-RNAi lines but higher in *OsATG8b*-OE lines than in ZH11 (Figure 7C), which shows that autophagy plays an important role at the grain-filling stage.

NHI is the main index of the efficiency of N distribution to grains and N grain filling (Guiboileau et al., 2012). The NHI of *OsATG8b*-RNAi was lower than that of ZH11, while that in *OsATG8b*-OE was higher (Figure 7D). As the NHI/HI ratio is considered a good indicator of NUE in plants (Masclaux-Daubresse and Chardon, 2011), we then measured the NHI/HI ratio of *OsATG8b*-RNAi, *OsATG8b*-OE, and ZH11. The results showed that the NHI/HI ratio increased dramatically in *OsATG8b*-OE lines but decreased in *OsATG8b*-RNAi lines when compared to ZH11 (Figure 7E). These data indicate that *OsATG8b*-mediated autophagy plays a role in grain NUE.

On the seventh day after ^15^NO_3_^–^ labeling, the ^15^N contents of *OsATG8b*-RNAi, *OsATG8b*-OE, and ZH11 showed no significant differences. This is consistent with the normal growth of the *OsATG8b-*RNAi and *OsATG8b*-OE lines under N-rich conditions (Supplementary Figure S7). The abundances of ^15^N in grains and remains were determined using isotopic ratio mass spectrometry, which enabled us to calculate the partitioning of ^15^N in grains (^15^NHI) by combining these values with DW and N% data. ^15^NHI and the ^15^NHI:HI ratio, an indicator for NRE, were lower in *OsATG8b*-RNAi lines and higher in *OsATG8b*-OE lines than in ZH11 (Figures 7F,G). Taken together, these ^15^N partitioning results show that *OsATG8b*-mediated autophagy significantly affects NRE during the grain-filling stage.

## Discussion

Plant autophagy plays important roles in growth and development, grain filling, response to pathogen infection and to abiotic and biotic stresses, and N recycling (Wada et al., 2009; Yoshimoto et al., 2009; Guiboileau et al., 2012). All of these functions have major agricultural relevance, and most *ATG* orthologs in crops have been identified in maize and rice (Chung et al., 2009; Xia et al., 2011). Here, we report that rice *OsATG8b* is involved in N recycling and thus affects rice yield and seed quality.

### *OsATG8b* Is a Functional Homologue of Yeast ScATG8 and a Useful Autophagosome Marker for Rice

Evolutionarily, autophagy is a highly conserved intracellular mechanism of degradation of cellular components in eukaryotic cells (Michaeli et al., 2016). At the elongation and final enclosure stages of the autophagosome, the linkage of ATG8 to PE anchors the former to both inner and outer membranes of the phagophore (Zientara-Rytter and Sirko, 2016). Therefore, the ATG8 protein is a useful molecular marker of autophagosomes, allowing for their distinction from other cellular vesicles and intracellular membranes (Yoshimoto et al., 2004; Zientara-Rytter and Sirko, 2016). Unlike yeast, which has a single *ATG8*, higher eukaryotes usually have an *ATG8* family. Rice has six *ATG8*s (Xia et al., 2011), and five of their proteins have the conservative glycine in the C-terminal for PE conjugation except for OsATG8f. OsATG8a, b, and c belong to subgroup I of the plant ATG8 phylogenetic tree (Supplementary Figure S1), as all three proteins have extra amino acids behind the conserved Gly residue and need cleavage by ATG4 to expose the Gly residue (Supplementary Figure S2). On the other hand, OsATG8d and e belong to subgroup II (Supplementary Figure S1), as both have an innate C-terminal-exposed Gly residue, which makes OsATG8 quickly proceed conjugate with PE without ATG4 processing (Supplementary Figure S2). Expression of nine *AtATG8* genes showed different patterns (Slavikova et al., 2005), which indicates that different *ATG8* members may have multiple non-redundant functions and that individual *ATG8*s may have specific functions.

Plant *ATG8s* can functionally complement yeast *atg8* mutants, such as those in *Arabidopsis* (Slavikova et al., 2005), soybean (Xia et al., 2012), and wheat (Pei et al., 2014). In our study, *OsATG8b* expression restored autophagy defects in the corresponding yeast *atg8* mutant (Figure 1). This indicated that OsATG8b has an autophagic function similar to yeast ATG8. At present, observation of GFP-ATG8 puncta has been shown to be the best and most convenient detection method for autophagic activity (Kliosnky, 2016). However, it is showed that GFP-ATG8 signal foci in cytoplasm might not be the true autophagosomes in the cytoplasm of *atg4a-1atg4b-1* double mutants (Yoshimoto et al., 2004) and *atg7-2* mutants (Suttangkakul et al., 2011) since the foci may be GFP-ATG8 aggregates (Kuma et al., 2007; Kim et al., 2012). However, in the presence of concanamycin A, the mutants (*atg7-2*, *atg5*, *atg10*, and *atg4a-1atg4b-1* in *Arabidopsis* and *atg7* in rice) always lack GFP-ATG8 labeled autophagic foci in the vacuole (Yoshimoto et al., 2004; Thompson et al., 2005; Phillips et al., 2008; Izumi et al., 2015). This indicates that vacuolar GFP-ATG8 spots should be utilized as an autophagy indicator instead of GFP-ATG8 dots (Chung, 2011). The sGFP-OsATG8b puncta in vacuoles of rice root cells in the presence of concanamycin A were observed (Figure 3B); therefore, sGFP-OsATG8b is considered to be a marker for measuring the autophagic activity of rice cells. We also detected autophagosomes in vacuoles of sGFP-ATG8b transgenic rice (Figure 3B). Free GFP released from fused sGFP-ATG8b also supports this transfer and accumulates in vacuoles (Supplementary Figure S4B). Therefore, the sGFP-ATG8b test is a biochemical way to monitor the autophagic flux of rice cells.

### *OsATG8b* Affects Grain Number and Grain Quality

*Arabidopsis* and maize *atg* mutants are sensitive to nutrient-limiting conditions (Hanaoka et al., 2002; Slavikova et al., 2005; Li et al., 2015). However, the *OsATG8b*-RNAi and *OsATG8b*-OE lines showed a relatively normal phenotype. In rice, there are six *ATG8s*, of which *OsATG8a*, *OsATG8b*, and *OsATG8c* have high homology. Data from RiceXpro (Supplementary Figure S8) showed that these three genes have similar expression patterns at vegetative stage. However, there are some different patterns during grain development; in particular, only *OsATG8b* expression increases with endosperm development (Supplementary Figure S8B). These data indicate that *OsATG8s* function redundantly in response to nutrient stress at the vegetative stages, but individual *ATG8*s may have specific functions in grain development. Indeed, in our study, *OsATG8b*-RNAi lines showed a chalky endosperm phenotype and carried small, loosely packed starch granules (Figures 6B,D), while in *OsATG8b-*OE lines endosperm, starch granules seemed larger and tighter (Figure 6D). Many genes and environmental factors control the grain endosperm chalkiness of rice (Siebenmorgen et al., 2013). Starch is the main storage material in rice grains, accounting for nearly 90% of the total dry weight, while protein accounts for about 8% of the endosperm weight of rice, filling the area between starch grains (Lin et al., 2014). Previous studies have shown that incomplete accumulation of starch and inadequate accumulation of proteins cannot fully fill the gap between starch granules, which may lead to the formation of chalk (Delrosario et al., 1968; Lin et al., 2014).

Starch and protein of rice grain are products of C and N, which are transported from source organs to produce starch and protein in precise quantities and proportions (Duan and Sun, 2005). C and N statuses are affected in *Arabidopsis atg* (*atg5* and *atg7*) mutants (Guiboileau et al., 2013; Masclaux-Daubresse, 2016). We showed that soluble protein content decreased in *OsATG8b*-RNAi lines and increased in *OsATG8b-*OE lines, while starch content showed no difference between these lines (Figures 6E,F). In *OsATG8b*-RNAi lines, autophagic activity was slightly inhibited, and grain yield and quality were reduced (Figures 4A,C). The root shortening phenotype in 7-day-old *OsATG8b*-RNAi seedling (Figures 4D,E) may be caused by this impaired grain, since there are no obvious morphological differences between *OsATG8b*-RNAi and ZH11 at other vegetative stages (Supplementary Figure S6). We deduced that knocking-down *OsATG8b* in grains may cause decreased degradation of stored proteins in the germinating grains and then attenuate the growth rate of roots at the grain germination stage. These results suggest that *OsATG8b*-RNAi lines produced chalky endosperm mainly by breaking the balance between C and N in rice grains.

During early reproductive stage, panicle primordia and spikelets differentiate and develop in the shoot apical meristems, and the top four leaves and their respective internodes are developed on the mature dwarf stem and leaves. All of these events are mainly maintained by the N storage in the epiphylls of the dwarf stem and supply of new N from soil (Yoneyama et al., 2016). Therefore, spikelet number is determined by the N obtained from both recycling from leaves and root uptake. Our data showed that grain number per plant in *OsATG8b*-OE lines increased while that in *OsATG8b*-RNAi lines decreased compared with that in ZH11 in the field, indicating that *OsATG8b*-mediated autophagy affects grain number mainly by influencing N recycling from the dwarf stem-attached leaves to spikelet development.

### *OsATG8b*-Mediated Autophagy Is Involved in N Recycling to Grains

Grain yield is affected by both soil N and remobilized N during reproductive stage (Kichey et al., 2007). To increase the NUE and crop yield, traditional methods focus on the operation of basic genes for N uptake and assimilation, such as *NRT*, *NR*, etc. (Pathak et al., 2008). In the grain-filling process, leaf organic N supply is more important because it contributes to plant N economy and limits the demand for exogenous N after flowering (Hanaoka et al., 2002). That is to say, the available N of grain was obtained from existing organic storage through recycling rather than from soil sources. Recently, studies on *Arabidopsis* and maize have shown that autophagy is the main factor affecting N recycling from senescent leaves to seeds (Guiboileau et al., 2012; Li et al., 2015). N recycling in senescent leaves was suppressed in *osatg7* at the vegetative stage, but the male sterility of *osatg7* limited evaluation of autophagy on both N economy and grain yield (Kurusu et al., 2014). Thus, we inferred that N recycling contributed by autophagy from the plant remains to grains in rice by over-expression and RNA interference of *OsATG8b*. Immunoblotting analysis results showed that autophagy activity is higher in *OsATG8b*-OE lines and a little lower in *OsATG8b*-RNAi than that in ZH11. Previous studies showed that OsATG8b antibody can also recognize OsATG8a and OsATG8c (Supplementary Figure S9). In *OsATG8b* RNAi lines, the band recognized by OsATG8b antibody represents the total OsATG8s, including OsATG8a, OsATG8b, and OsATG8c, so it is difficult to observe obvious differences in OsATG8b protein level with this method. Therefore, in our study, *OsATG8b*-RNAi lines showed slightly inhibited autophagic activity, which leads to reduced NRE from vegetative tissues to developing grains and finally results in reduced grain yield and quality. Meanwhile, reduced grain quality may cause decreased degradation of stored proteins in the germinating grains and then slow down the root growth at the grain germination stage. Conversely, *OsATG8b*-OE plants have higher yield and increased NRE (Figures 6, 7), and higher autophagic activity (Figure 4C). Thus, higher autophagic activity causes increased NRE, which leads to better grain yield. These results confirm that autophagy plays a crucial role in the N recycling process in rice. Therefore, improving N recycling by operating autophagy may be a useful strategy for increasing rice yield.

## Data Availability Statement

Data of this study are included in this article and its additional files. The material that supports the findings of this study is available from the corresponding author on request.

## Author Contributions

MZ and TF designed the research. TF, WY, XZ, XX, YX, and ML performed experiments. TF, MZ, XF, KX, and CT analyzed data. TF and MZ wrote the manuscript. All authors read and approved the final manuscript.

## Conflict of Interest

The authors declare that the research was conducted in the absence of any commercial or financial relationships that could be construed as a potential conflict of interest.

## Footnotes

1 https://ricexpro.dna.affrc.go.jp/

## Supplementary Material

FIGURE S1 Phylogenetic tree of ATG8s by amino sequence alignment of different species. *Glycine max* (Gm), *Arabidopsis thaliana* (At), *Saccharomyces cerevisiae* (Sc), *Selaginella moellendorffii* (Sm), *Oryza sativa* (Os), *Homo sapiens* (Hs), *Solanum lycopersicum* (Sly), *Triticum aestivum* (Ta), and *Brachypodium distachyon* (Bd). Deduced amino acid sequences were aligned by CLUSTALX; the phylogenetic tree was generated by the neighbor-joining method and constructed using MEGA4.

FIGURE S2 Alignment of ATG8 amino acid sequence and 3D model of OsATG8b. **(A)** Alignment of ATG8 amino acid sequences from rice, *Arabidopsis*, human, and yeast. Arrows indicate the C-terminal glycine residue, which is processed by ATG4 cysteine protease. Residues constituting W- and L-sites are colored red and green, respectively. Sc, *S*. *cerevisiae*; Hs, *Homo sapiens*; At, *Arabidopsis thaliana*; Os, *Oryza sativa*. **(B)** 3D models of OsATG8b. Two hydrophobic pockets responsible for the recognition of Trp and Leu are labeled W-site and L-site, respectively, and circled.

FIGURE S3 Subcellular localization of sGFP-OsATG8b in rice protoplasts. Bars = 1 μm.

FIGURE S4 Immunoblot detection of the vacuolar delivery of GFP in *GFP-OsATG8b* lines and immunoblot analysis with OsATG8b antibodies. Total proteins extracted from shoots of 14-day-old-seedlings in *GFP-OsATG8b* (OE) and *GFP* (G1) transgenic lines and ZH11. **(A)** Total proteins were subjected to immunoblot analysis with GFP antibody. **(B)** OsATG8b antibodies recognize the endogenous proteins OsATG8(a/b/c) as well as the GFP fusion proteins in ZH11 and *GFP-OsATG8b* transgenic lines.

FIGURE S5 The expression of *OsATG8a* and *OsATG8c* in ZH11, *OsATG8b*-OE, and *OsATG8b*-RNAi lines. **(A)** Sequence comparison with other homologous genes for construction of *OsATG8b* RNAi. The RNAi fragment is demarcated by the box. **(B,C)** qRT-PCR analysis of *OsATG8a* and *OsATG8c* expression. The seedlings of ZH11, *OsATG8b-*OE, and *OsATG8b*-RNAi at four-leaf stage were divided into the shoots and roots. *OseEF-1a* was used as an internal reference. Error bars indicate standard deviations of independent biological replicates (*n* = 3). No asterisks mean no significant difference (*t*-test).

FIGURE S6 *OsATG8b*-RNAi (Ri) and *OsATG8b*-OE (OE) lines exhibited a relatively normal phenotype and a similar growth rate when compared with ZH11 at 30 and 60-day after germination (DAG). **(A,B)** Phenotype of *OsATG8b*-RNAi and *OsATG8b*-OE plants grown under low (LN, 0.2 mM NH_4_NO_3_) **(A)** and high N contents (HN, 5 mM NH_4_NO_3_) **(B)** at 30 DAG. **(C,D)** Statistical analysis of root **(C)** and shoot **(D)** length of *OsATG8b*-RNAi and *OsATG8b*-OE plants grown under both LN and HN conditions at 30 DAG. **(E,F)** Phenotype of *OsATG8b*-RNAi and *OsATG8b*-OE plants grown under LN **(E)** and HN **(F)** conditions at 60 DAG. **(G,H)** Statistical analysis of root **(G)** and shoot **(H)** length of *OsATG8b*-RNAi and *OsATG8b*-OE plants grown under both LN and HN conditions, at 60 DAG. Three biological replicates, each containing thirty plants, were used for data analysis. Error bars indicate standard deviations of independent biological replicates. No asterisks mean no significant difference (*t*-test).

FIGURE S7 ^15^N-labeling efficiency of ZH11, *OsATG8b*-RNAi (Ri), and *OsATG8b*-OE (OE) lines. Plants at 40-day after germination were labeled for 5-day with ^15^NO_3_^–^, harvested 7-day later, and then assayed for ^15^N content in seedlings. Results are the mean ± SD from three plants. Three biological replicates, each containing three plants, were used for data analysis.

FIGURE S8 Spatio-temporal expression of *OsATG8a* **(A)**, *OsATG8b* **(B)**, and *OsATG8c* **(C)** in various tissues/organs throughout the entire plant growth in the field. Data were obtained from RiceXpro (http://ricexpro.dna.affrc.go.jp/).

FIGURE S9 OsATG8b antibody cannot distinguish OsATG8a, OsATG8b, and OsATG8c. The proteins of OsATG8a, OsATG8b, and OsATG8c were expressed in *E. coli*, and detected by the anti-OsATG8b antibody.

TABLE S1 All primers used in this study.

## Abbreviations

APE1: aminopeptidase 1
CVT: cytoplasm-to-vacuole targeting
DAG: days after germination
DW: dry weight
HI: harvest index
IRRI: International Rice Research Institute
LSCM: laser-scanning confocal microscopy
mAPE1: mature APE1
N: nitrogen
NHI: nitrogen harvest index
NUE: nitrogen use efficiency
NRE: nitrogen recycling efficiency
OE: over-expression
ORF: open reading frame
*OsATG8b*: *Oryza sativa* autophagic-related gene 8b
PE: phosphatidylethanolamine
qRT-PCR: quantitative real-time PCR
RNAi: RNA interference.
