# Supplementary figures and images for: A Rice Autophagy Gene OsATG8b Is Involved in Nitrogen Remobilization and Control of Grain Quality
Source: Front Plant Sci. 2020 Jun 4;11:588. doi: 10.3389/fpls.2020.00588 (PMC7287119; doi:10.3389/fpls.2020.00588)

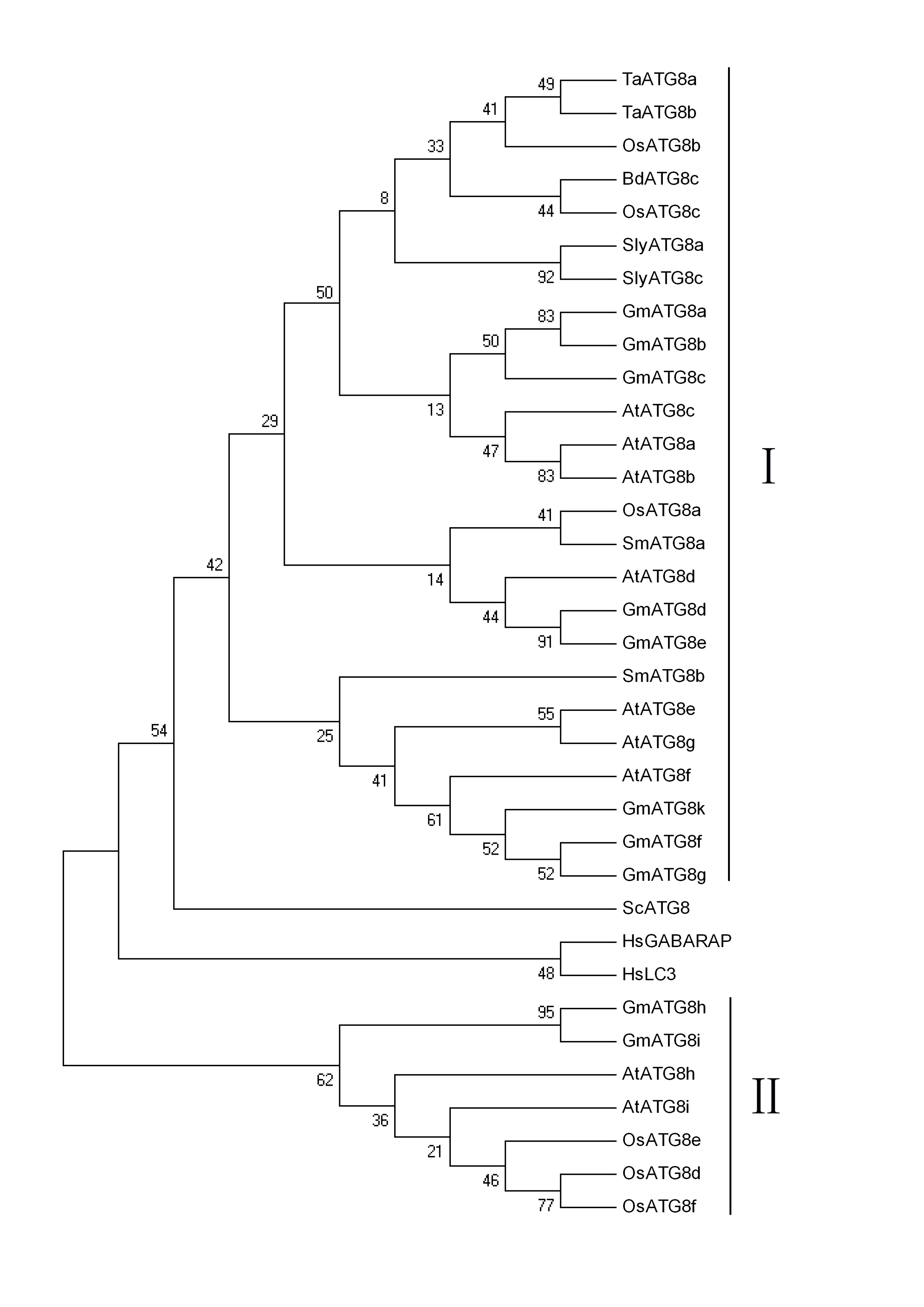

Supplement: FIGURE S1 — Phylogenetic tree of ATG8s by amino sequence alignment of different species. Glycine max (Gm), Arabidopsis thaliana (At), Saccharomyces cerevisiae (Sc), Selaginella moellendorffii (Sm), Oryza sativa (Os), Homo sapiens (Hs), Solanum lycopersicum (Sly), Triticum aestivum (Ta), and Brachypodium distachyon (Bd). Deduced amino acid sequences were aligned by CLUSTALX; the phylogenetic tree was generated by the neighbor-joining method and constructed using MEGA4. [file Image_1.TIF]

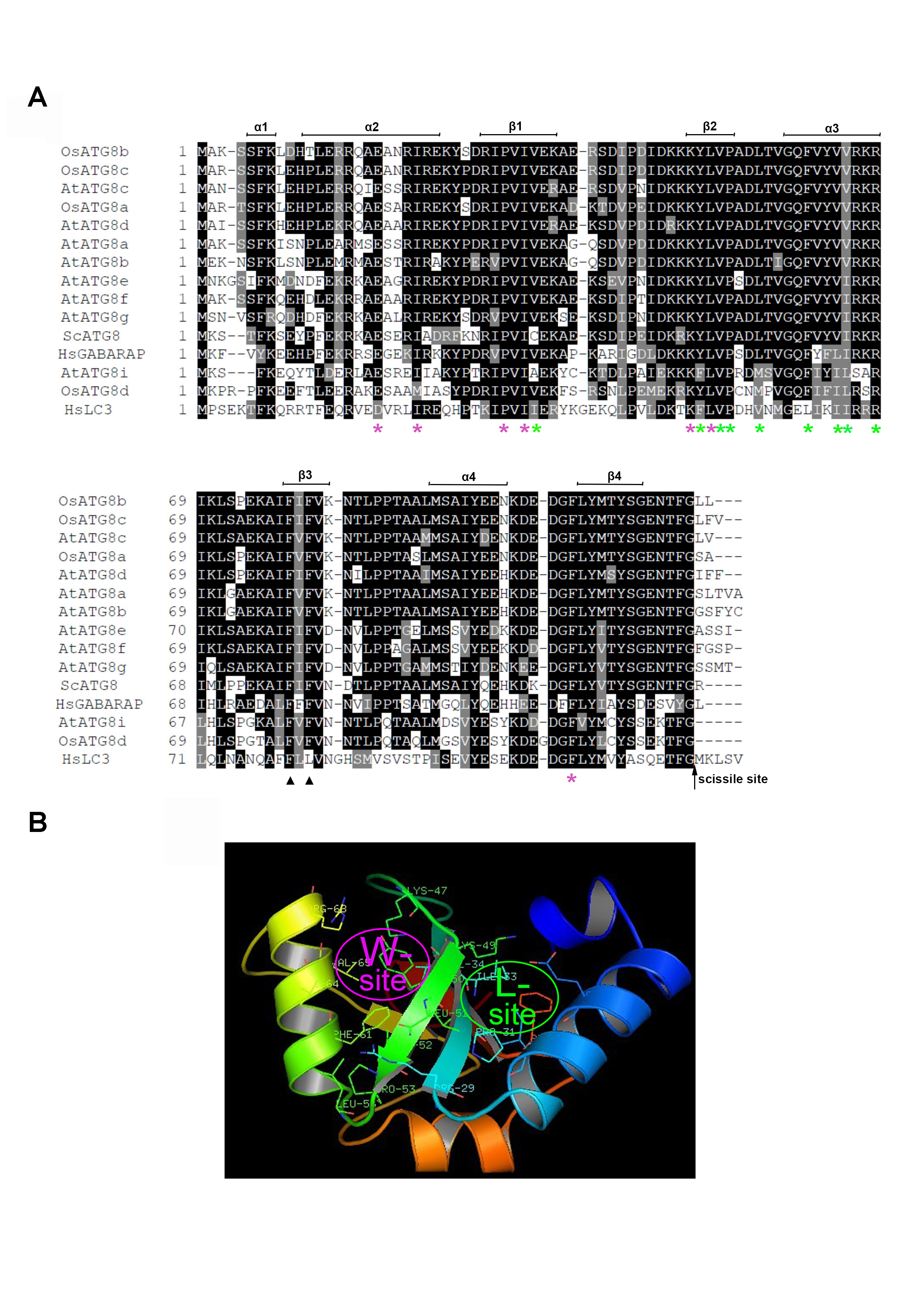

Supplement: FIGURE S2 — Alignment of ATG8 amino acid sequence and 3D model of OsATG8b. (A) Alignment of ATG8 amino acid sequences from rice, Arabidopsis, human, and yeast. Arrows indicate the C-terminal glycine residue, which is processed by ATG4 cysteine protease. Residues constituting W- and L-sites are colored red and green, respectively. Sc, S. cerevisiae; Hs, Homo sapiens; At, Arabidopsis thaliana; Os, Oryza sativa. (B) 3D models of OsATG8b. Two hydrophobic pockets responsible for the recognition of Trp and Leu are labeled W-site and L-site, respectively, and circled. [file Image_2.TIF]

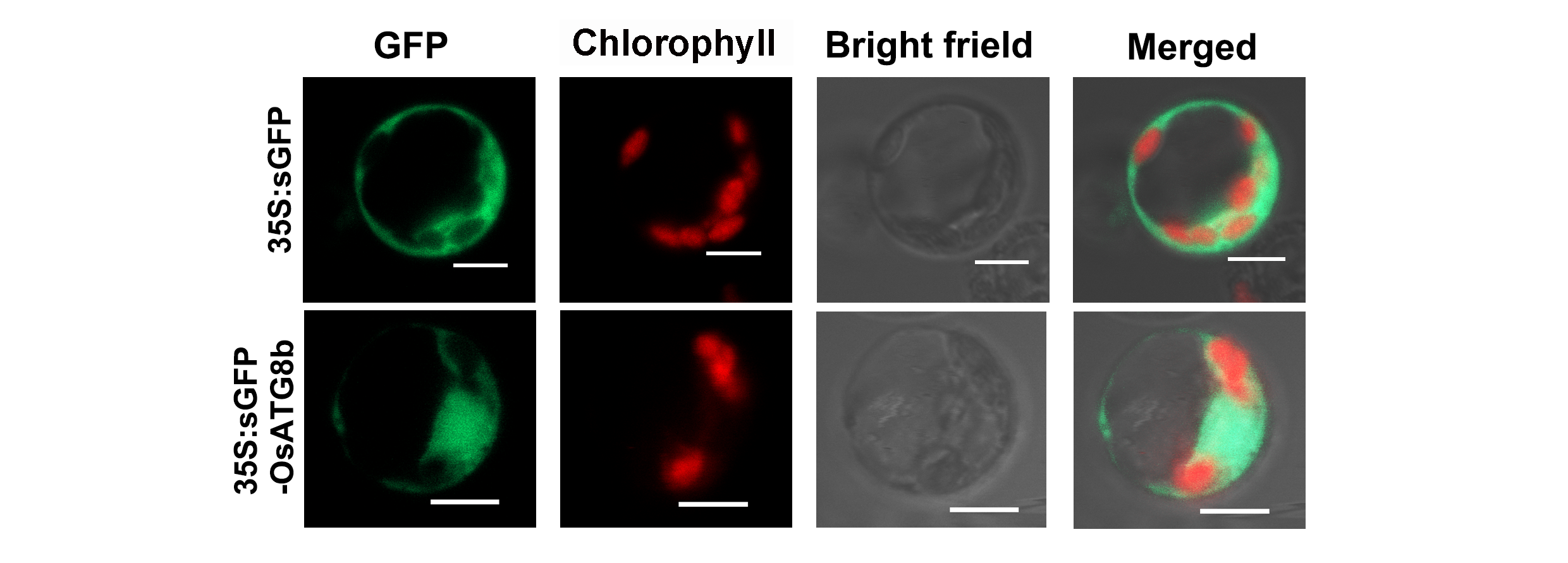

Supplement: FIGURE S3 — Subcellular localization of sGFP-OsATG8b in rice protoplasts. Bars = 1 μm. [file Image_3.TIF]

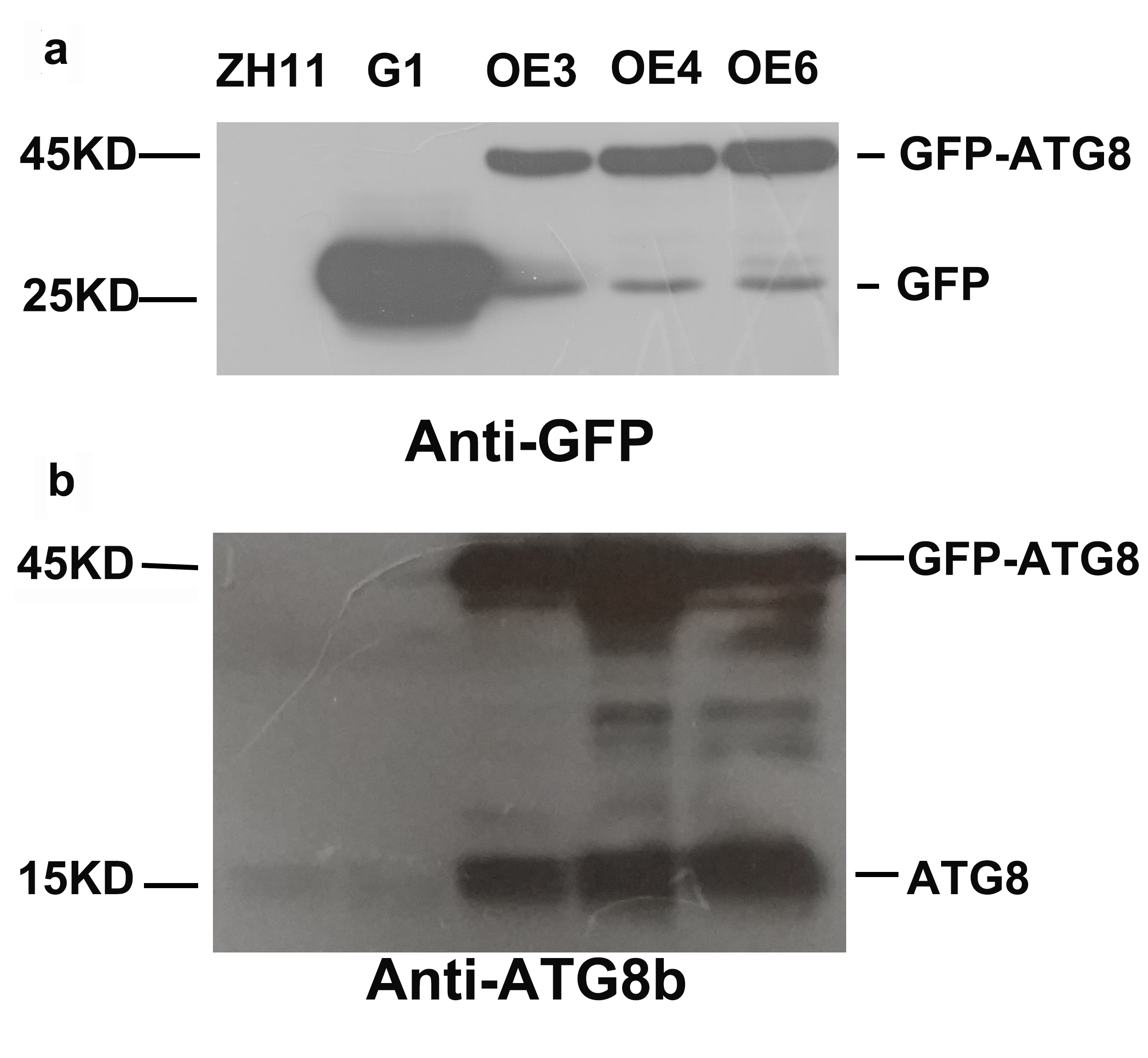

Supplement: FIGURE S4 — Immunoblot detection of the vacuolar delivery of GFP in GFP-OsATG8b lines and immunoblot analysis with OsATG8b antibodies. Total proteins extracted from shoots of 14-day-old-seedlings in GFP-OsATG8b (OE) and GFP (G1) transgenic lines and ZH11. (A) Total proteins were subjected to immunoblot analysis with GFP antibody. (B) OsATG8b antibodies recognize the endogenous proteins OsATG8(a/b/c) as well as the GFP fusion proteins in ZH11 and GFP-OsATG8b transgenic lines. [file Image_4.TIF]

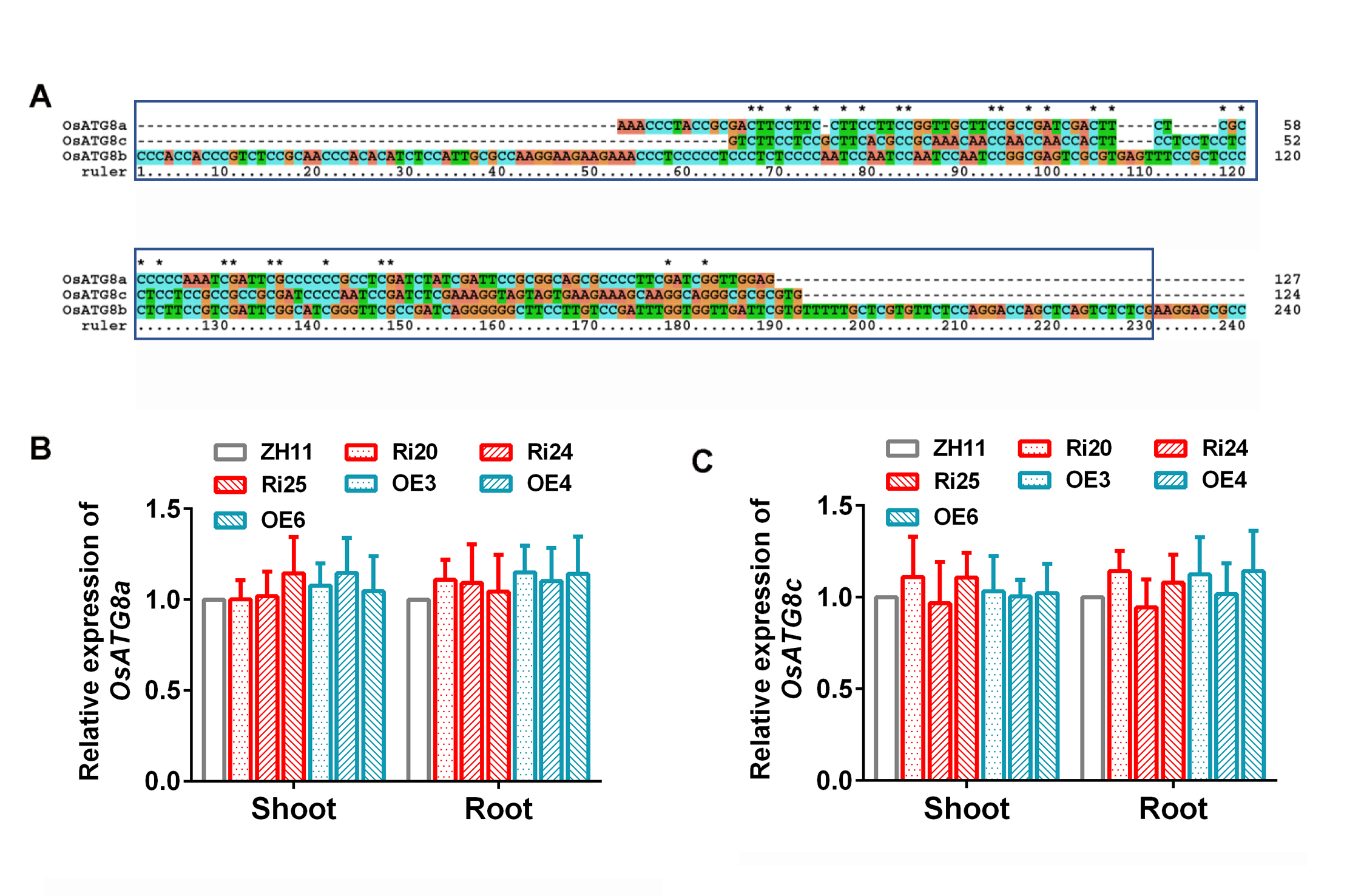

Supplement: FIGURE S5 — The expression of OsATG8a and OsATG8c in ZH11, OsATG8b-OE, and OsATG8b-RNAi lines. (A) Sequence comparison with other homologous genes for construction of OsATG8b RNAi. The RNAi fragment is demarcated by the box. (B,C) qRT-PCR analysis of OsATG8a and OsATG8c expression. The seedlings of ZH11, OsATG8b-OE, and OsATG8b-RNAi at four-leaf stage were divided into the shoots and roots. OseEF-1a was used as an internal reference. Error bars indicate standard deviations of independent biological replicates (n = 3). No asterisks mean no significant difference (t-test). [file Image_5.TIF]

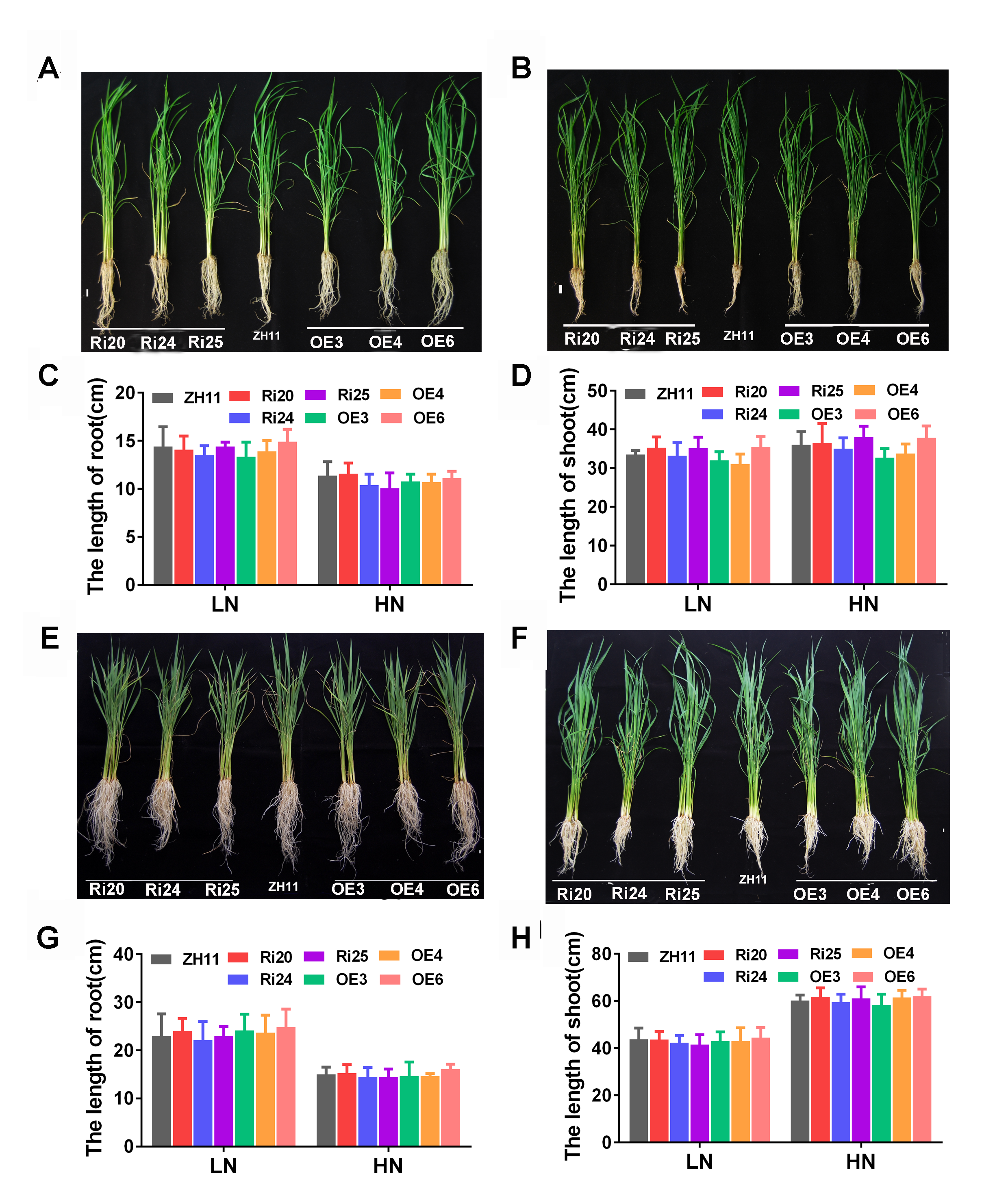

Supplement: FIGURE S6 — OsATG8b-RNAi (Ri) and OsATG8b-OE (OE) lines exhibited a relatively normal phenotype and a similar growth rate when compared with ZH11 at 30 and 60-day after germination (DAG). (A,B) Phenotype of OsATG8b-RNAi and OsATG8b-OE plants grown under low (LN, 0.2 mM NH4NO3) (A) and high N contents (HN, 5 mM NH4NO3) (B) at 30 DAG. (C,D) Statistical analysis of root (C) and shoot (D) length of OsATG8b-RNAi and OsATG8b-OE plants grown under both LN and HN conditions at 30 DAG. (E,F) Phenotype of OsATG8b-RNAi and OsATG8b-OE plants grown under LN (E) and HN (F) conditions at 60 DAG. (G,H) Statistical analysis of root (G) and shoot (H) length of OsATG8b-RNAi and OsATG8b-OE plants grown under both LN and HN conditions, at 60 DAG. Three biological replicates, each containing thirty plants, were used for data analysis. Error bars indicate standard deviations of independent biological replicates. No asterisks mean no significant difference (t-test). [file Image_6.TIF]

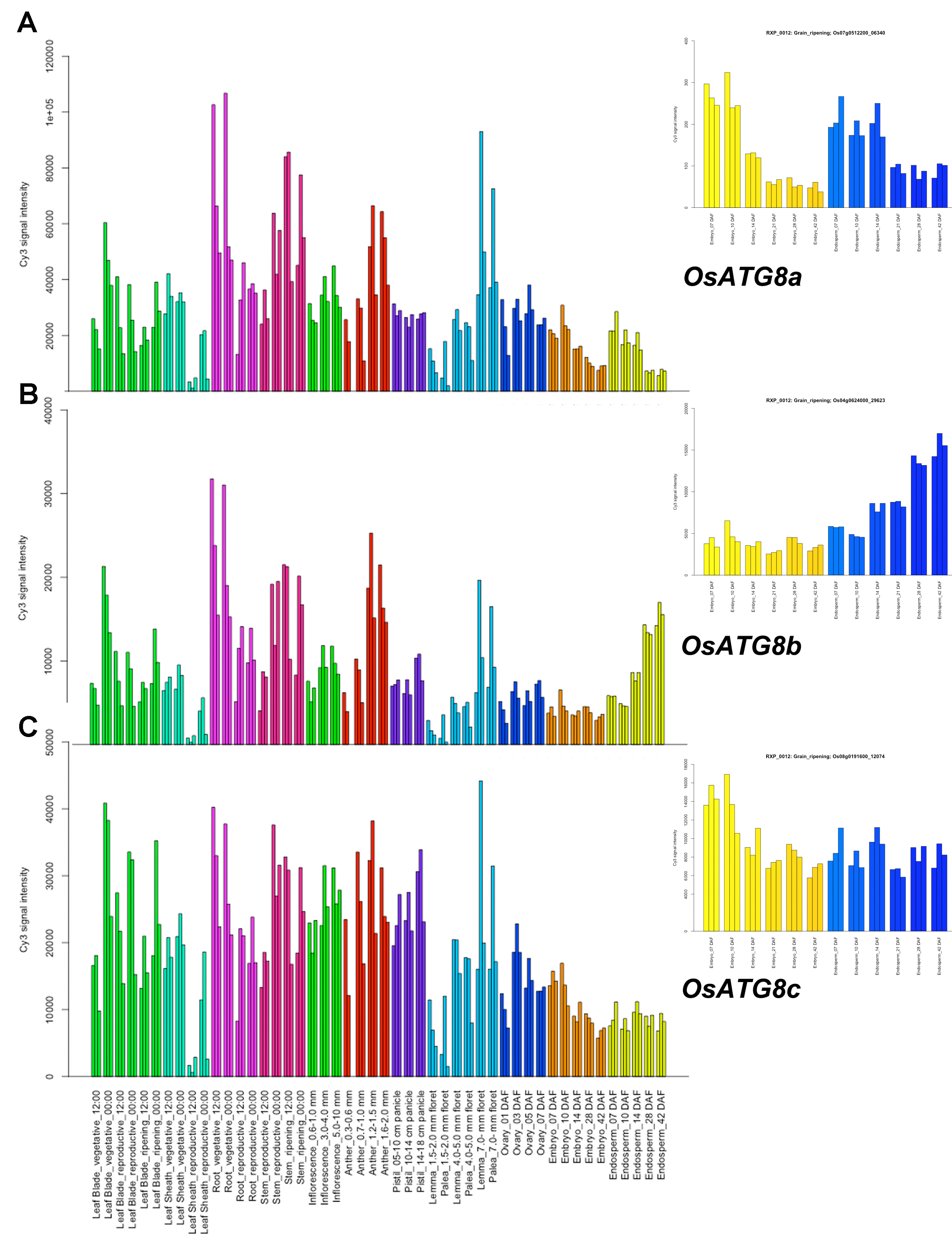

Supplement: FIGURE S8 — Spatio-temporal expression of OsATG8a (A), OsATG8b (B), and OsATG8c (C) in various tissues/organs throughout the entire plant growth in the field. Data were obtained from RiceXpro (http://ricexpro.dna.affrc.go.jp/). [file Image_8.JPEG]

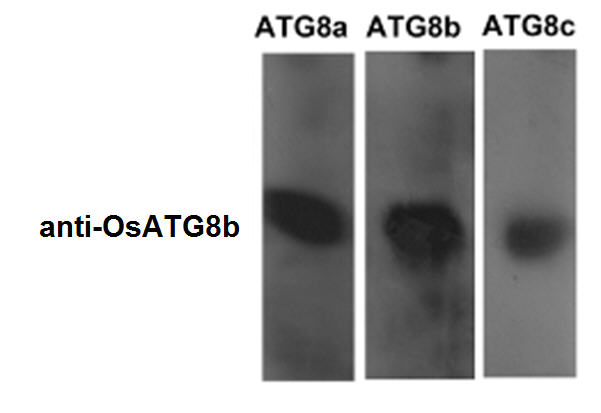

Supplement: FIGURE S9 — OsATG8b antibody cannot distinguish OsATG8a, OsATG8b, and OsATG8c. The proteins of OsATG8a, OsATG8b, and OsATG8c were expressed in E. coli, and detected by the anti-OsATG8b antibody. [file Image_9.TIF]
