# Supplementary material for: A Rice Autophagy Gene OsATG8b Is Involved in Nitrogen Remobilization and Control of Grain Quality
Source: Front Plant Sci. 2020 Jun 4;11:588. doi: 10.3389/fpls.2020.00588 (PMC7287119; doi:10.3389/fpls.2020.00588)
Supplement: TABLE S1 — All primers used in this study. [file Table_1.DOC]

**Table S1. List of the primers in this study.**

**qRT-PCR primers**

| **Name** | **Sequences(5’-3’)** |
| --- | --- |
| *eEF-1α*-F | GCACGCTCTTCTTGCTTTC |
| *eEF-1α*-R | AGGGAATCTTGTCAGGGTTG |
| *OsATG8b*-F | GCTCCTTGTCCGATTTGGTG |
| *OsATG8b*-R | TGTTAGCCTCAGCCTGCCTC |
| *OsATG8a*-F | ACTAGTCCTTCCTTCCGGTTGCTTC |
| *OsATG8a*-R | TTACGCCTGCTTGCCTCCTTTCCAG |
| *OsATG8c*-F | CGGCAAGAACACGCTTCCACCAAC |
| *OsATG8c*-R | CGGGCGACCTATTTACACGAGGCATT |

**Vector construction primers**

| **Name** | **Sequences(5’-3’)** |
| --- | --- |
| Yeast com-F | ATGGATCCTGATGGCCAAGAGCTCGTTC |
| Yeast com-R | GCTTCTAGACTAGAGCAGCCCAAAGGTG |
| Yeast GFP-F | TGGATCCTGATGGCCAAGAGCTCGTTC |
| Yeast GFP-R | GCTTCTAGACTAGAGCAGCCCAAAGGTG |
| *OsATG8b* OE-F | ACTCCATGGACATGGTGAGCAAGGGCGAG |
| *OsATG8b* OE-R | AGTGGTTACCCTAGAGCAGCCCAAAGGTG |
| *OsATG8b* RNAi-F | TAGGTACCACTAGTCCCACCACCCGTGTCCG |
| *OsATG8b* RNAi-R | ATGGATCCGAGCTCCGAGAGACTGAGCTGG |
